# Spatial Organization of Fish Assemblages and Its Relation to Environmental Factors in the Lower Yalong River, China

**DOI:** 10.1002/ece3.71922

**Published:** 2025-08-07

**Authors:** Huijun Ru, Xiangxiang Wu, Bin Xu, Yan Zhang, Fan Wu, Baoshan Ma

**Affiliations:** ^1^ National Agricultural Science Observing and Experimental Station of Chongqing Yangtze River Fisheries Research Institute, Chinese Academy of Fishery Science Wuhan China

**Keywords:** fish assemblages, lower Yalong River, plateau species, spatial variations, stream order

## Abstract

Based on data collected from 31 sampling sites during June and August 2018 in the three ordered rivers (the tributaries of the Anning River, the Anning River, and the lower Yalong River), fish assemblages in the region were investigated regarding their spatial variations and their relationship with environmental factors. Totally, 48 species were collected, with 47 being native and one being exotic. Among them, one was classified as endangered. Fish species diversity increased first and then decreased, reaching its peak in the mid‐order river. Obvious variations were found in fish assemblages depending on the stream order. There were three site groups: one covering the tributaries of the Anning River, one covering the Anning River, and one covering the lower Yalong River. From low‐ to high‐order rivers, the alterations of key species within the fish communities exhibited a turnover pattern. The fish assemblages in the low‐order river were primarily dominated by Nemacheilida, whereas the mid‐order river and the high‐order river displayed a dominance of Gobioninae and Cultrinae, respectively. These spatial variations might be primarily impacted by water depth and turbidity. Moreover, this study highlights the critical role of mid‐order rivers in sustaining fish diversity within dendritic stream networks of mountain rivers.

## Graphical abstract

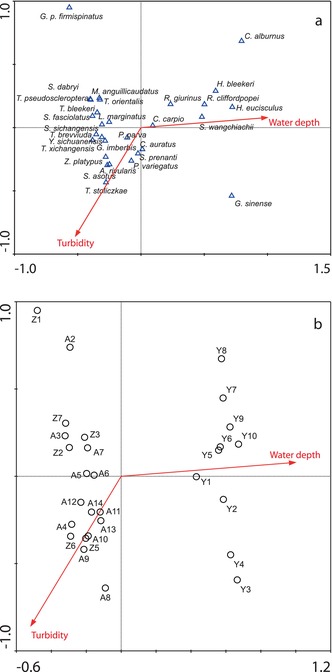

## 1 Introduction

Fish assemblages are influenced by various environmental factors, including historical, biotic, and abiotic factors of river ecosystems (Jackson et al. 2001; Nilsson et al. 2005; Araújo et al. 2009). For abiotic factors, habitat variables including altitude, channel width, water temperature, substrate size, and velocity differed among differently ordered rivers, significantly affecting the distribution of river fish assemblages owing to diverse physiological demands and habitat selection among different species (Yan et al. 2011; Li et al. 2016; Zhu et al. 2017; Chen et al. 2019).

Riverine fish communities can be impacted by aquatic habitat availability among diverse spatial scales and the movement capacity of individuals across the habitats (Thornbrugh and Gido 2010; Ma et al. 2024). Thus, measures of local fish community structure can be estimated through species composition in neighboring drainage basins, and the positions inside the drainage (Schaefer and Kerfoot 2004; Chen et al. 2019). Many authors have proposed that there are spatial dependencies of fish assemblage structure across river habitats (such as varying‐sized rivers and diverse geological basins). Fausch et al. (2002) put forward the riverscapes perspective, suggesting the viewing of dendritic stream networks in the whole interconnected network structure rather than in disjunct parts. Grant et al. (2007) further suggested that dendritic network structure was vital for shaping biotic assemblages, and emphasized the importance of movement among branches and the heterogeneous habitats associated with a branch‐node structure.

It is crucial to understand the fish assemblage structure within river network ecosystems in community ecology, providing valuable information for ecological restoration and biodiversity conservation (Araújo et al. 2009). Moreover, the nature of continuities between mainstream and tributaries, and among tributaries, probably causes spatial auto‐correlation of ecological processes, biotic and abiotic factors in a watershed at the river network scale (Grant et al. 2007). The location of tributaries inside the drainage network can determine local fish communities, as well as their movement and extinction (Grenouillet et al. 2004; Yan et al. 2011; Li et al. 2016; Zhu et al. 2017; Chen et al. 2019). Dendritic stream networks involve the sharp transition in stream size at tributary confluences, and these habitats may be crucial for the regulation of biota exchange across different branches in such networks (Benda et al. 2004; Lowe et al. 2006; Grant et al. 2007). Certain species probably prefer respective habitats depending on stream size; however, to utilize resources and take refuge, numerous stream organisms may migrate across mainstem and tributary habitats in diverse life stages (Thornbrugh and Gido 2010).

The abiotic and biotic environmental factors of the lower Yalong River, the Anning River, and its tributaries vary considerably (Ma et al. 2021). Therefore, this is an ideal area to study the variation of fish assemblage structures among differently ordered rivers. The Yalong River Basin, the largest tributary of the Jinsha River, is located in the southern Qinghai‐Tibet Plateau and is rich in hydropower resources. Currently, five cascades in the lower Yalong River have been completed, the river environment has significantly changed, and most of the lower Yalong River forms the reservoir areas (Ma et al. 2021). Relative to free‐flowing stream segments, dam‐created impoundments are characterized by wider and deeper water bodies, slower flows, as well as smaller substrates (Tiemann et al. 2004; Gillette et al. 2005; Wang et al. 2013). These changes in local habitats probably change fish communities by reducing lotic species numbers while increasing lentic species numbers (Gillette et al. 2005; Yang et al. 2011; Wang et al. 2013; Liu et al. 2022).

The Anning River Basin, the largest tributary of the lower Yalong River, exhibits a topographical gradient, being lower in the eastern and southern regions while higher in the western and northern areas, with altitudes along the mainstream varying from approximately 900 to 2400 m. Its mid‐downstream is characterized by deep waters, broad channels, few boulders, and moderate‐flowing water (Ma et al. 2021). Conversely, the tributaries of the Anning River feature narrow channels, high altitudes, fast‐flowing water, shallow waters, and abundant boulders (Ma et al. 2023). Previous research indicated that fish assemblages in the Anning River were more significantly affected by natural conditions than by hydropower projects (Ma et al. 2023). As the mid‐order river, the Anning River exerts a critical role in sustaining the ecosystem structure and function of the Yalong River (Ma et al. 2021). The mid‐domain effect, which assumes that spatial boundaries cause more overlap of species' ranges towards the center of an area, has been extensively adopted for explaining the unimodal diversity pattern with elevation (Chen et al. 2019). In addition, many researchers also pointed out that fish species diversity usually increases depending on stream size, peaking in the mid‐elevation reaches of rivers (Roberts and Hitt 2010; Sui et al. 2014; Zhang et al. 2020). Thus, we assumed that mid‐order rivers, that is, the Anning River, exhibit the highest fish diversity.

This study tested fish specimens and environmental factors from 31 sites in the lower Yalong River, the Anning River, and their tributaries at the altitudes of 2311–991 m. The study aimed to (i) investigate spatial variations in fish species composition, dietary guilds, and fish assemblages across differently ordered rivers in the lower Yalong River; (ii) test the hypothesis that mid‐order rivers exhibit the highest fish diversity; and (iii) identify key environmental factors that influence fish assemblages.

## 2 Materials and Methods

### 2.1 Study Area

The Yalong River Basin, a tributary of the Jinsha River, is located in the southern Qinghai‐Tibet Plateau. Originating from the southern of the Bayan Har Mountains in Qinghai Province, it flows southeast, entering Sichuan Province, passes through Garze and Liangshan Prefecture, and finally flows into the Jinsha River at Panzhihua City. The reach below the Litang estuary is the lower Yalong River, with an average altitude of about 2500 m (Bao 2014; Xiong et al. 2024). The lower Yalong River is situated in a subtropical humid climate zone and a warm temperate zone, with abundant rainfall and dense forests (Wang 2014). Due to the great topographic height difference and large north–south latitude variation, the characteristics of the plane and vertical variation are formed, which lead to the complex climatic conditions in the basin. The average annual temperature in the lower Yalong River is 18°C–21°C (Xiong et al. 2024). The climate type based on the subtropical zone has distinct drought and rainy seasons, and the annual precipitation is 900–1300 mm, mostly in summer and autumn. Water resources are extremely rich, which are mainly comprised of rainfall, groundwater, and snowmelt water (Bao 2014).

### 2.2 Sample Collection Timing and Sites

**FIGURE 1:**
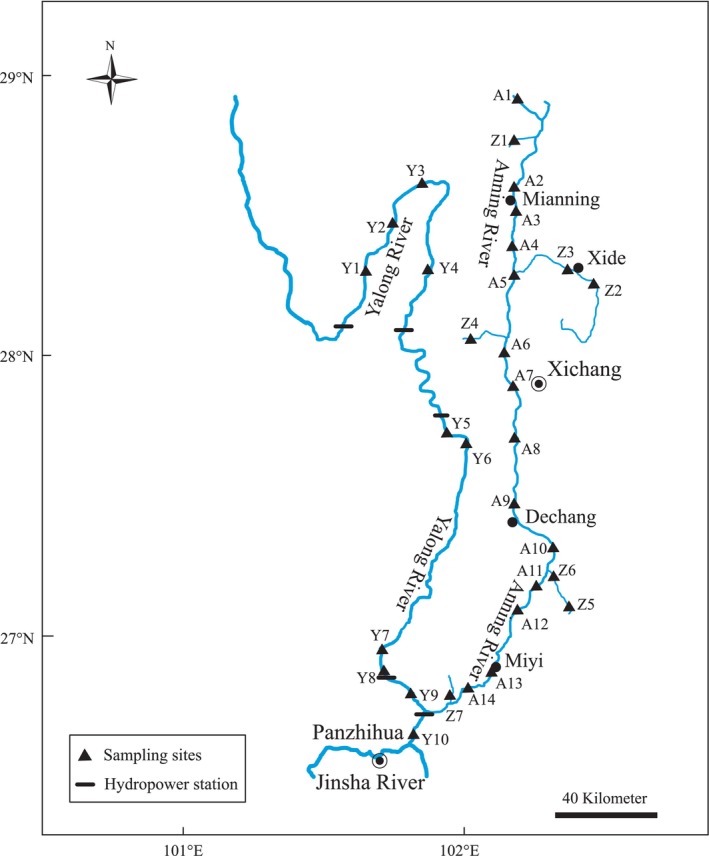
Sampling sites in the lower Yalong River and its tributaries. The tributaries of the Anning River, 7 sites (Z1‐Z7); the Anning River, 14 sites (A1‐A14); the lower Yalong River, 10 sites (Y1‐Y10).

The 31 sites were surveyed from June to August 2018, covering the lower Yalong River, the Anning River, and its tributaries (Figure 1), with elevations showing a reduction from 2311 to 991 m. Apart from habitat types, we also considered access when selecting sampling sites (Liu, Li, et al. 2021). Among them, there were 7 in the tributaries of the Anning River (low‐order river), 14 sites in the mainstream of the Anning River, and 10 sites in the mainstream of the lower Yalong River (high‐order river) (Figure 1).

### 2.3 Field Sampling

During June and August 2018, fish species were sampled through electrofishing, set gill nets (mesh size 3–15 cm), and trap nets (mesh size 1–1.5 cm) among different habitats. The sampling length of electrofishing varied from 1 to 2 km. Set gill nets and trap nets were placed in situ for around 15 h. Additionally, the river water depth, current velocity, and channel width were considered to determine the range of river sampling (0.5–1 km), net number, and mesh size. By capturing fishes, they were subjected to a species‐level assessment (Zhang et al. 2019), including weight (g) and total length (mm) of fishes. Recognizable fishes were released in situ, while nonrecognizable ones were saved in 7% formaldehyde to be subsequently identified in the laboratory. Based on main feeding categories, fishes were assigned to dietary guilds (Zhang et al. 2019). The habitat preference of each species was classified based on personal observation and scientific literature (Zhang et al. 2019; Liu, Wang, et al. 2021).

### 2.4 Environmental Factor Determination

Longitude, latitude, and altitude (m) were measured with the Garmin GPS‐76 system. Five transects were employed to compute water depths (m). The laser rangefinder was used to measure channel widths (m) at every site in the beginning, middle, and end, and the average value was taken. Flow velocity (m/s) was measured in the middle of each site with a direct reading flow meter (Global water FP‐311), whereas transparency (cm) was explored with a Secchi disc. Water temperature (°C), pH, conductivity (μs/cm), turbidity (NTU), and dissolved oxygen (mg/L) were determined with the EXO2 Water Quality Sonde (YSI Inc., USA). Environmental factors were measured three times at 50 cm beneath the surface and 3 m away from margins in large and mainstream tributaries (Araújo et al. 2009). In some small tributaries and upstream, these factors were determined at half of the river depth and width from three points. Substratums were classified as (1) boulder (> 256 mm), (2) cobble (64–256 mm), (3) pebble + gravel (2–64 mm), (4) sand (0.06–2 mm), and (5) silt + clay (< 0.06 mm). Therefore, in accordance with Barbour et al.'s criteria (1999), the substratum type proportion at each site was evaluated (Jiang et al. 2013).

### 2.5 Data Analysis

Fish species diversity was determined by Margalef richness index (*d*), Shannon‐Wiener diversity index (*H′*), and Pielou evenness index (*J*) as follows (Belaoussoff et al. 2003): *d* = (*S* − 1)/ln*N*, *H*′ = −∑*P*_*i*_ (ln*P*_*i*_), and *J* = *H*′/ln*S*, where *S* indicates the species number, *N* is the total individual number, and *P*_*i*_ refers to the individual percentage of species *i*.

The fish assemblage similarity degree across different sampling sites was analyzed by cluster analysis (the group average hierarchical sorting method) according to the Bray–Curtis similarity matrix (Liu, Li, et al. 2021). Relative abundance data matrices were employed to determine the Bray–Curtis similarity coefficient. Subsequently, analysis of similarity (ANOSIM) was performed to assess different fish assemblages among different site groups. Key species that made great contributions to maintaining community structure were identified through similarity percentage analysis (SIMPER), with a cumulative contribution rate of community similarity within a group being around 90%. Statistical analysis was performed using “CLUSTER”, “ANOSIM”, and “SIMPER” modules in PRIMER v6 (Clarke and Warwick 2001).

Nonparametric test was employed to analyze spatial variations of fish species diversity and environmental parameters among differently ordered rivers. Post hoc comparison among differently ordered rivers was conducted by Kruskal‐Wallis test in the presence of significance. Associations among diverse environmental factors were analyzed by Spearman's correlation analysis. Factors without significant spatial variations (*p* > 0.05) or those closely related to other factors (correlation coefficients > 0.6) were excluded (Liu, Li, et al. 2021). These above analyses were conducted using R software.

Only fish species with relative abundance > 0.3% were included in the subsequent analysis. The associations between fish communities and environmental factors were analyzed by constrained canonical ordinations. Canoco software showed the unimodal model (gradient length = 5.509). Thus, Canonical Correspondence Analysis (CCA) was adopted for plotting the relations of fish communities/sampling sites with environmental factors (Lepš and Šmilauer 2003) To obtain normality, environmental factors were transformed with log (x + 1) (except for pH) or Asin (Sqrt(x)). The threshold of alpha = 0.05 was applied to select explanatory variables for incorporation in the model through forward selection. CANOCO 5.0 was applied to perform statistical analysis (Ter Braak and Smilauer 2012).

## 3 Results

### 3.1 Species Composition

During June and August 2018, a total of 3544 individuals belonging to 48 fish species were obtained, including 47 native and one exotic species. All individuals fell in 12 families, 5 orders. Cypriniformes served as the predominant order, occupying 75% (36 species), followed by Siluriformes (6 species) and Perciformes (4 species). Cyprinidae was the dominant family, which encompassed 21 species, followed by Cobitidae (14 species), Siluridae (2 species), Bagridae (2 species), and Gobiidae (2 species). Balitoridae, Amblycipitidae, Sisoridae, Oryziatidae, Synbranchidae, Eleotridae, and Callichthyidae each contained one species (Table 1). There were a total of 9 native species native to the upper Yangtze River, with one being considered endangered ( *Percocypris pingi* ) (Jiang et al. 2016).

**TABLE 1:** *N*% of fish species collected in the lower Yalong River and its tributaries during June and August 2018.

Species	TA	MA	MY	Trophic guild	Habitat preference
Cypriniformes	97.56	92.61	76.88		
Cyprinidae	27.14	80.01	76.34		
Danioninae	0.78	0.64	0.00		
1. *Zacco platypus*	0.78	0.64	0.00	Piscivore	Genera
Arosudae	0.00	0.06	0.00		
2. *Ctenopharyngodon idella*	0.00	0.06	0.00	Herbivore	Lentic
Cultrinae	0.00	1.09	44.62		
3. *Hemiculter leucisculus*	0.00	0.19	25.27	Omnivore	Lentic
4. *Hemiculter bleekeri*	0.00	0.90	8.06	Omnivore	Lentic
5. *Hemiculter tchangi*	0.00	0.00	3.23	Omnivore	Lentic
6. *Culter alburnus*	0.00	0.00	8.06	Piscivore	Genera
Hypophthalmichthyinae	0.00	0.06	0.00		
7. *Hypophthalmichthys molitrix*	0.00	0.06	0.00	Planktivore	Lentic
Gobioninae	14.65	67.54	1.08		
8. *Hemibarbus maculatus*	0.00	0.06	0.00	Insectivore	Genera
9. *Pseudorasbora parva*	0.28	4.88	1.08	Omnivore	Genera
10. *Gnathopogon imberbis*	12.21	12.85	0.00	Insectivore	Genera
11. *Abbottina rivularis*	2.16	49.74	0.00	Omnivore	Genera
Acheilognathinae	0.00	0.19	0.00		
12. *Rhodeus sinensis*	0.00	0.19	0.00	Planktivore	Genera
Barbinae	0.00	0.00	4.30		
13. *Spinibarbus sinensis*	0.00	0.00	2.15	Omnivore	Genera
14. *Percocypris pingi*	0.00	0.00	2.15	Piscivore	Lotic
Labeoninae	0.28	0.13	0.00		
15. *Garra pingi*	0.28	0.13	0.00	Periphytivore	Lotic
Schizothoracinae	10.71	1.41	23.66		
16. *Schizothorax wangchiachii*	1.22	1.41	18.28	Periphytivore	Lotic
17. *Schizothorax prenanti*	2.44	0.00	3.23	Periphytivore	Lotic
18. *Schizothorax kozlovi*	0.11	0.00	2.15	Insectivore	Lotic
19. *Gymnocypris potanini firmispinatus*	6.94	0.00	0.00	Insectivore	Lotic
Cyprininae	0.72	8.87	2.69		
20. *Cyprinus carpio*	0.55	1.48	1.61	Omnivore	Lentic
21. *Carassius auratus*	0.17	7.39	1.08	Omnivore	Lentic
Balitoridae	5.33	2.63	0.00		
22. *Sinogastromyzon sichangensis*	5.33	2.63	0.00	Periphytivore	Lotic
Cobitidae	65.09	9.96	0.54		
Nemacheilinae	64.21	7.20	0.54		
23. *Paracobitis variegatus*	2.94	0.39	0.54	Insectivore	Lotic
24. *Schistura dabryi*	4.50	0.00	0.00	Insectivore	Lotic
25. *Schistura fasciolatus*	12.32	0.06	0.00	Insectivore	Lotic
26. *Trilophysa bleekeri*	11.82	2.12	0.00	Omnivore	Lotic
27. *Triplophysa orientalis*	9.99	0.06	0.00	Omnivore	Lotic
28. *Trilophysa brevviuda*	4.27	0.71	0.00	Omnivore	Lotic
29. *Triplophysa pseudoscleroptera*	0.83	0.06	0.00	Omnivore	Lotic
30. *Triplophysa stoliczkae*	7.21	2.06	0.00	Omnivore	Lotic
31. *Triplophysa xichangensis*	4.77	0.13	0.00	Omnivore	Lotic
32. *Triplophysa stenura*	0.00	0.06	0.00	Omnivore	Lotic
33. *Triplophysa* sp.	0.00	0.06	0.00	Omnivore	Lotic
34. *Yunnanilus sichuanensis*	5.55	1.48	0.00	Insectivore	Lotic
Cobitinae	0.89	2.76	0.00		
35. *Misgurnus anguillicaudatus*	0.89	2.31	0.00	Omnivore	Genera
36. *Paramisgurnus dabryanus*	0.00	0.45	0.00	Omnivore	Genera
Siluriformes	2.44	4.18	15.05		
Siluridae	0.55	2.63	0.54		
37. *Silurus asotus*	0.55	2.63	0.00	Piscivore	Genera
38. *Silurus meridionalis*	0.00	0.00	0.54	Piscivore	Genera
Bagridae	0.00	0.26	1.08		
39. *Leiocassis crassilabris*	0.00	0.13	1.08	Piscivore	Genera
40. *Pseudobagrus emarginatus*	0.00	0.13	0.00	Piscivore	Genera
Amblycipitidae	0.00	0.90	0.00		
41. *Liobagrus marginatus*	0.00	0.90	0.00	Piscivore	Lotic
Sisoridae	1.89	0.39	13.44		
42. *Glyptothorax sinense*	1.89	0.39	13.44	Insectivore	Lotic
Cyprinodontiformes	0.00	0.19	0.00		
Oryziatidae	0.00	0.19	0.00		
43. *Monopterus albus*	0.00	0.19	0.00	Planktivore	Genera
Synbranchiformes	0.00	0.06	0.00		
Synbranchidae	0.00	0.06	0.00		
44. *Oryzias latipes*	0.00	0.06	0.00	Piscivore	Genera
Perciformes	0.00	2.96	8.06		
Eleotridae	0.00	0.39	0.00		
45. *Micropercops swinhonis*	0.00	0.39	0.00	Insectivore	Genera
Gobiidae	0.00	2.51	6.99		
46. *Rhinogobius giurinus*	0.00	1.93	4.84	Insectivore	Genera
47. *Rhinogobius cliffordpopei*	0.00	0.58	2.15	Insectivore	Genera
Callichthyidae	0.00	0.06	1.08		
48. *Oreochromis* sp.	0.00	0.06	1.08	Planktivore	Genera

Abbreviations: MA, the mainstream of the Anning River; MY, the mainstream of the lower Yalong River; TA, the tributaries of the Anning River.

In spatial terms, 25 species were obtained in the tributaries of the Anning River, whereas 39 and 19 species were sampled from the Anning River and the lower Yalong River, respectively. The fish assemblages in the tributaries of the Anning River were dominated by Nemacheilida, whereas those in the Anning River and the lower Yalong River exhibited a dominance of Gobioninae and Cultrinae, respectively.

### 3.2 Fish Species Diversity

**FIGURE 2:**
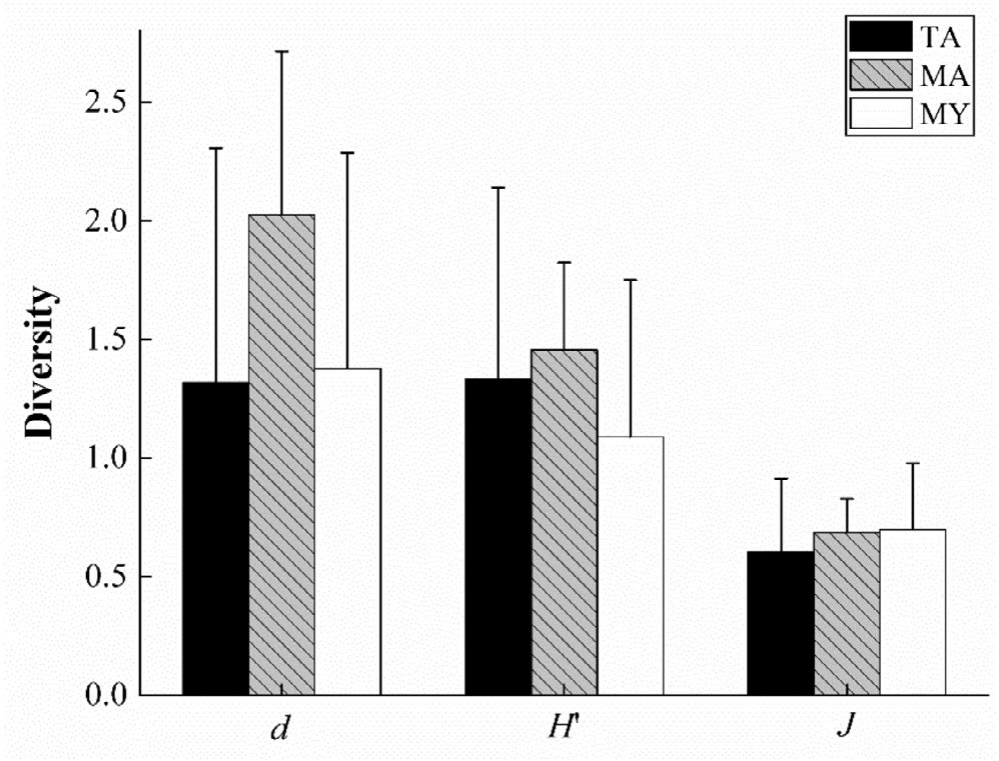
Fish species diversity in the lower Yalong River and its tributaries. TA, the tributaries of the Anning River; MA, the mainstream of the Anning River; MY, the mainstream of the lower Yalong River.

No significant differences were observed in all of the three fish diversity indices among differently ordered rivers (*p* > 0.05). The Margalef richness index (*d*) in the Anning River was higher than that in the lower Yalong River and the tributaries of the Anning River. The Anning River exhibited the highest Shannon‐Wiener diversity index (*H′*), followed by the tributaries of the Anning River, while the lower Yalong River showed the lowest value. The Pielou evenness index (*J*) of various ordered rivers was similar to one another (Figure 2).

### 3.3 Fish Assemblages

Totally, 48 fish species were divided into 6 trophic guilds, namely, insectivore, periphytivore, omnivore, piscivore, planktivore, and herbivore (Table 1). The omnivorous species was the predominant guild in the whole basin of the lower Yalong River (19 species), followed by the insectivorous guild (11 species); the conditions in the Anning River and its tributaries, and the lower Yalong River were similar (Table 2). Piscivores were usually observed in the Anning River (6 species) and the lower Yalong River (4 species), but seldom found in the tributaries of the Anning River (2 species). Herbivorous species ( *Ctenopharyngodon idella* ) were only detected in the Anning River. The predominant guild was lotic species in the tributaries of the Anning River, whereas the percentage of lentic species was the highest in the lower Yalong River (Table 3).

**TABLE 2:** Species richness of trophic guilds in the lower Yalong River and its tributaries.

River	Number of species					
	Insectivore	Periphytivore	Omnivore	Piscivore	Planktivore	Herbivore
TA	7	4	12	2		
MA	8	3	17	6	4	1
MY	5	2	7	4	1	
Whole basin	11	4	19	9	4	1

Abbreviations: MA, the mainstream of the Anning River; MY, the mainstream of the lower Yalong River; TA, the tributaries of the Anning River.

**TABLE 3:** Species richness of habitat preference in the lower Yalong River and its tributaries.

River	Number of species		
	Lotic	Genera	Lentic
TA	17	6	2
MA	16	17	6
MY	6	8	5
Whole basin	21	20	7

Abbreviations: MA, the mainstream of the Anning River; MY, the mainstream of the lower Yalong River; TA, the tributaries of the Anning River.

**FIGURE 3:**
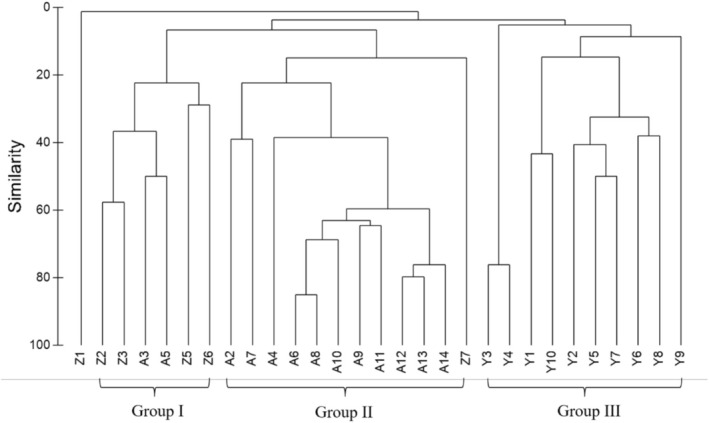
Classification of fish assemblages in the lower Yalong River and its tributaries based on the Bray–Curtis similarity of fish species at 29 sites.

According to cluster analysis during June and August 2018 concerning fish species relative abundance data, 29 sampling sites (no fish was caught at A1 and Z4) were divided into three site‐groups. The sites of the Anning River and its tributaries were separated from the Yalong sites. Most tributary sites (except for Z1 and Z7) were included in Group I, most Anning sites (except for A3 and A5) were assigned to Group II, whereas every Yalong site was assigned to Group III (Figure 3). Fish assemblages were classified following the stream order splitting pattern. Upon ANOSIM, differences among the three site‐groups were of statistical significance (Global *R* = 0.680, *p <* 0.01).

**FIGURE 4:**
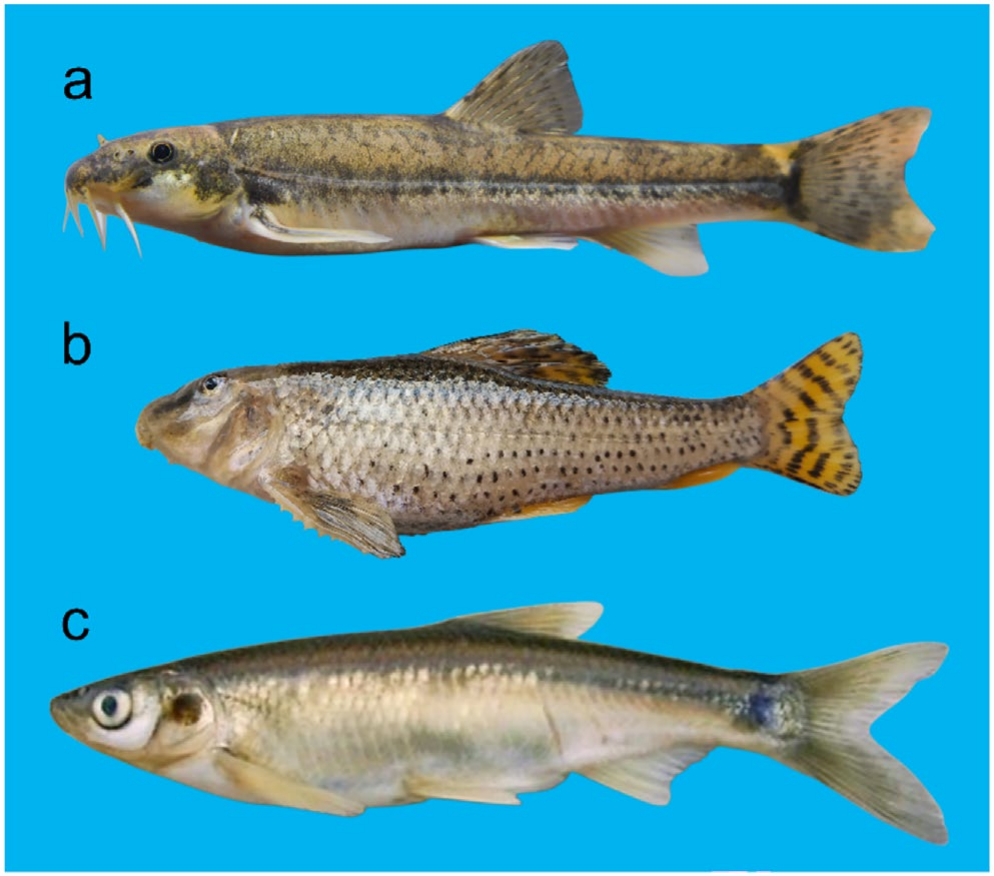
Key fish species in the lower Yalong River and its tributaries. (a) *Trilophysa bleekeri* in the tributaries of the Anning River; (b) *Abbottina rivularis* in the Anning River; (c) *Hemiculter leucisculus* in the lower Yalong River.

According to SIMPER analysis, key species contributing to intra‐group fish community similarity across differently ordered rivers were identified. In the tributaries of the Anning River, the key species included *Trilophysa bleekeri*, *Triplophysa orientalis* , *Gymnocypris potanini firmispinatus*, *Trilophysa brevviuda*, and *Paracobitis variegatus* . In the Anning River, *Abbottina rivularis* , *Carassius auratus* , *Gnathopogon imberbis* , and *Pseudorasbora parva* exerted a significant role, while the key species were *Hemiculter leucisculus* , *Rhinogobius giurinus* , *Glyptothorax sinense*, *Schizothorax wangchiachii* , and *Hemiculter bleekeri* in the lower Yalong River (Table 4 and Figure 4). From low‐order (the tributaries of the Anning River) to high‐order (the lower Yalong River) rivers, the changes in key species were manifested in “species loss” and “species gain” processes, which showed the turnover pattern.

**TABLE 4:** Key species among differently ordered rivers in the lower Yalong River.

	Tributaries of the Anning River			Mainstream of the Anning River			Mainstream of the lowerYalong River		
	AA	AS	Cum	AA	AS	Cum	AA	AS	Cum
*Trilophysa bleekeri*	30.53	15.15	63.68						
*Triplophysa orientalis*	12.95	2.15	72.74						
*Gymnocypris potanini firmispinatus*	19.54	1.84	80.49						
*Trilophysa brevviuda*	5.71	1.37	86.26						
*Paracobitis variegatus*	5.4	1.03	90.6						
*Abbottina rivularis*				43.08	30.34	73.9			
*Carassius auratus*				6.54	2.28	79.46			
*Gnathopogon imberbis*				8.39	2.08	84.52			
*Pseudorasbora parva*				5.36	1.89	89.11			
*Hemiculter leucisculus*							19.82	8.31	48.21
*Rhinogobius giurinus*							7.95	2.18	60.86
*Glyptothorax sinense*							17.22	2.12	73.12
*Schizothorax wangchiachii*							16.24	1.81	83.62
*Hemiculter bleekeri*							6.31	1.37	91.59

Abbreviations: AA, average abundance (%); AS, average similarity; Cum, cumulative contribution.

### 3.4 Relations Between Fish Assemblages and Environmental Factors

Water depth, channel width, current velocity, conductivity, dissolved oxygen, pH, turbidity, and the proportion of boulders were significantly different among differently ordered rivers (*p* < 0.05, Table 5). With the increase in stream order, the current velocity and proportion of boulders decreased, while the water depth, channel width, conductivity, and dissolved oxygen increased. Specifically, the water depth, channel width, conductivity, and dissolved oxygen of the lower Yalong River significantly increased in comparison with the Anning River (*p* < 0.05). The Anning River had the highest turbidity, followed by the tributaries of the Anning River, and the lower Yalong River exhibited the lowest value (*p* < 0.05). The proportion of boulders was the highest in the tributaries of the Anning River, followed by the Anning River, and the lower Yalong River (*p* < 0.05). Altitude, water temperature, the proportions of cobbles, pebbles + gravels, sands, and silts + clays were not significantly different among differently ordered rivers (*p* > 0.05).

**TABLE 5:** Environmental parameters among differently ordered rivers in the lower Yalong River.

	Tributaries of the Anning River	Mainstream of the Anning River	Mainstream of the lower Yalong River
Altitude (m)	1706.26 ± 479.03	1473.4 ± 322.96	1287.38 ± 217.03
Water depth (m)	0.58 ± 0.18^b^	0.79 ± 0.25^b^	37.04 ± 10.13^a^
Channel width (m)	32.75 ± 28.41^b^	95.65 ± 66.49^b^	306.08 ± 285.36^a^
Transparency (cm)	> 24.61 ± 20.37	21.28 ± 23.49	63.47 ± 63.31
Current velocity (m/s)	1.3 ± 0.28^a^	1.07 ± 0.35^ab^	0.74 ± 0.45^b^
Water temperature (°C)	16.93 ± 4.07	18.69 ± 3.2	19.49 ± 2.85
Dissolved oxygen (mg/L)	7.73 ± 0.3^b^	7.76 ± 0.46a^b^	8.73 ± 1.00^a^
pH	8.27 ± 0.23^ab^	8.11 ± 0.2^b^	8.42 ± 0.23^a^
Conductivity (μS/cm)	93.32 ± 53.42^b^	121.47 ± 60.23^b^	185.63 ± 19.28^a^
Turbidity (NTU)	186.66 ± 231.78^ab^	289.86 ± 265.89^a^	48.57 ± 62.37^b^
Boulder (%)	45.00 ± 25.00^a^	28.93 ± 22.38^ab^	16.00 ± 16.47^b^
Cobble (%)	18.57 ± 6.90	24.64 ± 13.93	23.50 ± 18.27
Pebble plus gravel (%)	17.57 ± 8.44	20.36 ± 13.37	19.50 ± 15.71
Sand (%)	12.43 ± 9.55	13.64 ± 11.78	31.00 ± 27.67
Silt plus clay (%)	6.43 ± 11.07	12.43 ± 14.68	10.00 ± 14.91

*Note:* Values having different superscripts in each line are significantly different (*p* < 0.05) from each other.

**FIGURE 5:**
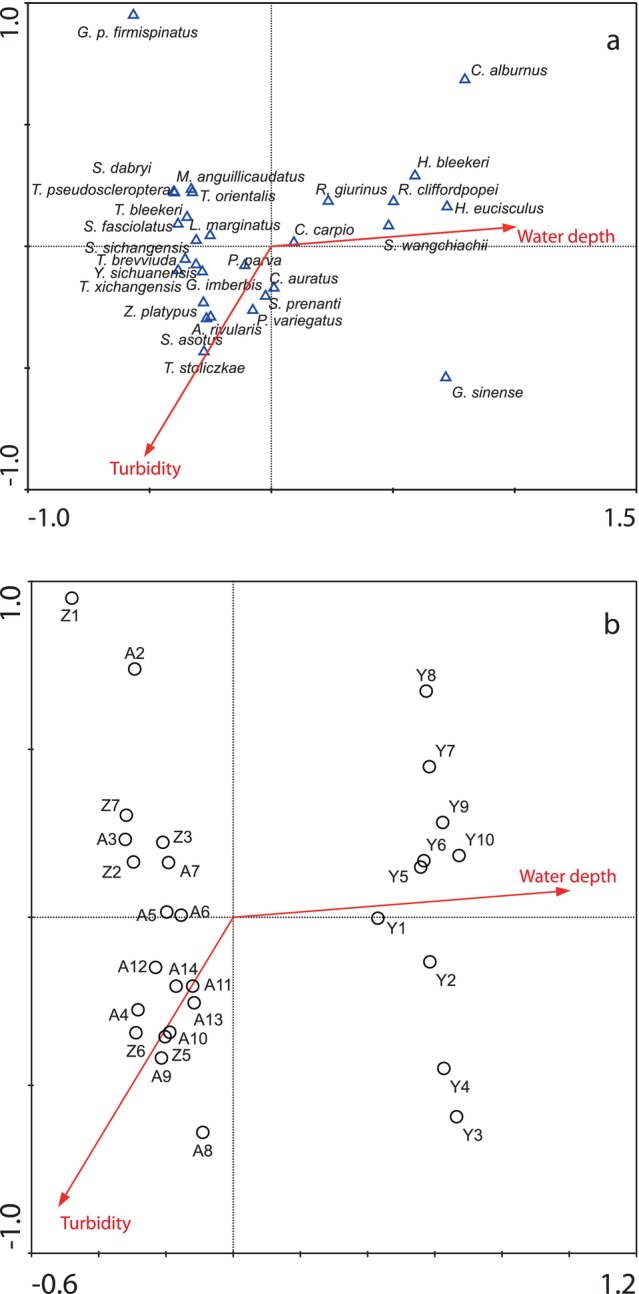
Canonical correspondence analysis (CCA) ordination plots depicting relationships between fish assemblages and environmental variables (a), and sampling sites and environmental variables (b) in the lower Yalong River and its tributaries.

Through Spearman's correlation analysis, water depth was positively related to channel width and conductivity and negatively associated with the proportion of boulders (Table S1). Therefore, after eliminating not spatially differentiated and associated factors, five factors, including water depth, turbidity, dissolved oxygen, pH, and current velocity, were used for initial CCA. Afterwards, upon forward selection, only water depth and turbidity were kept (Table 6), which interpreted 17.7% of total variation of species composition, and water depth interpreted the highest variation (11.3%), followed by turbidity (6.4%). In accordance with CCA ordination plots, Nemacheilinae was the dominant plateau subfamily, which preferred shallower water depth and usually appeared in the tributaries of the Anning River (Figure 5a). Furthermore, some species, including *Trilophysa bleekeri*, *Paracobitis variegatus* , and *Gymnocypris potanini firmispinatus*, preferred habitats with shallower water depth. Certain species, including *Pseudorasbora parva* and *Abbottina rivularis* , preferred moderate water depth, typically found in the Anning River. On the contrary, some species preferred slow‐flowing water, like *Culter alburnus* , *Hemiculter leucisculus* , *Glyptothorax sinense*, and *Hemiculter bleekeri* , and were dominant in the lower Yalong River, among which *Culter alburnus* , *Hemiculter leucisculus* , and *Hemiculter bleekeri* preferred habitats with deeper water depth (Figure 5a). *G*. *p*. *firmispinatus* preferred water with low turbidity, while *Silurus asotus* has relatively low requirements for water quality, with high turbidity (Figure 5a).

**TABLE 6:** Percentage of variance explained by the environmental variables used in the Canonical Correspondence Analysis (CCA) in the Yalong River and its tributaries.

Variables	% Explained	Contribution %	Pseudo‐*F*	*p*	*p*_adj_
Water depth	11.3	38.7	3.4	0.001	0.003
Turbidity	6.4	21.9	2.0	0.012	0.030
pH	4.9	16.8	1.6	0.050	0.083
Dissolved oxygen	3.7	12.6	1.2	0.255	0.319
Current velocity	2.9	10.0	0.9	0.521	0.521

Sampling sites were classified into two groups according to water depth and turbidity (Figure 5b). One group included every Yalong site characterized by deeper water depth, whereas the other one included every site of the Anning River and its tributaries and was featured by shallower water depth. The classification results basically conform to the cluster results on the basis of fish assemblages. Different from the clustering results of fish community structure, the difference between the mainstream and tributaries of the Anning River was not obvious.

## 4 Discussion

### 4.1 Fish Assemblage Differences Among Differently Ordered Rivers

Local habitat and tributary spatial location variables significantly affect fish assemblages (Thornbrugh and Gido 2010; Li et al. 2016; Zhu et al. 2017; Chen et al. 2019). For landscapes, rivers are considered as the connected networks that have the definable “network geometry”, with stream order being dominant (Liu, Lin, et al. 2021). Biotic and abiotic river characteristics alter from low‐order streams to high‐order locations. Lower‐order streams with higher altitude usually have lower water temperature, higher current velocity, shallower water, and narrower channels (Murugavel and Pandian 2000). Therefore, landscape location in the river network geometry may exert an influence on fish species distribution and diversity (Liu, Lin, et al. 2021). Fish species diversity usually increases depending on stream size, peaking in the mid‐elevation reaches of rivers (Roberts and Hitt 2010; Sui et al. 2014; Zhang et al. 2020). Our results indicated that local fish species diversity (both Margalef richness index and Shannon‐Wiener diversity index) increased to reach a maximum at the mid‐order river, that is, the Anning River. As the greatest tributary in the lower Yalong River, the aquatic biodiversity of the Anning River exerts a vital impact on maintaining ecosystem structure and function in the Yalong River (Ma et al. 2021).

Large landscape locations, such as stream size, stream order, and link magnitude have been identified as vital factors for shaping stream fish assemblage structure (Liu, Lin, et al. 2021). In this study, fish communities displayed significant variation with stream order. The similar result was found in the fish assemblages in the Ren River basin, which also significantly altered depending on the stream order (Liu, Lin, et al. 2021). Lin et al. (2025) identified significant differences in fish community structures between the mainstream and tributaries of the upper Jinsha River. The mainstream was primarily inhabited by *Schizothorax* and *Ptychobarbus* species, whereas tributary communities were dominated by *Schizopygopsis* and *Triplophysa* species. Thornbrugh and Gido (2010) observed the slightly reduced variability and declined dissimilarity to the mainstream as the distance from the Kansas River increased in fish assemblages. Zhu et al. (2017) pointed out that compared with land use, local habitat and tributary spatial location exerted a significant influence on fish assemblages in the Qingyi River. According to Chen et al. (2019), fish community structure in different orders of the Xin'an River was different. From first‐ to third‐order rivers, there may be changes in key species in their fish communities and revealed a nested pattern, whereas the changes of key species in the fish community exhibited a turnover pattern from third‐ to fifth‐order rivers. In this study, from low‐order to high‐order rivers, the changes of key species were manifested in “species loss” and “species gain” processes, showing a turnover pattern.

### 4.2 Environmental Impacts on Fish Assemblages

Various environmental factors can influence fish assemblages (Wang et al. 2019; Liu, Li, et al. 2021). At a river network scale, fluvial systems are significantly different in stream volume and size, habitat complexity and diversity, as well as local habitat profiles, like current velocity and flow regime (Zhu et al. 2017; Chen et al. 2019). Owing to the physiological, behavioral, and habitat preference heterogeneities among species (Jackson et al. 2001), local fish assemblages are associated with stream segment habitat factors, including altitude (Ma et al. 2023), water temperature (Wang et al. 2003), and substrate size (Wang et al. 2013; Ma et al. 2023). Certain stream size indicators, like water depth (Harvey and Stewart 1991), channel width (Yan et al. 2010), and discharge (Chu et al. 2015), have been found to determine local fish diversity. In this study, water depth and turbidity are vital environmental factors affecting fish assemblages in the lower Yalong River. Along the continuum from low‐order river (e.g., tributaries of the Anning River) to high‐order river (e.g., the lower Yalong River), shifts in key species occur through distinct processes of “species loss” and “species gain,” reflecting a clear turnover pattern without species overlap. Consequently, fish turnover patterns across rivers of different orders are strongly associated with altitude and habitat characteristics such as water depth and turbidity.

Turbidity, a quantitative indicator reflecting the concentration of suspended substances in water, is closely associated with fish habitat selection (Pyron et al. 2019; del Puerto et al. 2022; Severo‐Neto et al. 2023). During the summer flood season, increased upstream inflow introduces sediments and fine particles from both the mainstream and tributaries, leading to elevated turbidity levels in the Anning River (Table 5, Ma et al. 2021). Turbidity exerts both direct and indirect effects on fish distribution (del Puerto et al. 2022). It is also correlated with dissolved oxygen levels, potentially impacting species with specific oxygen requirements. Moreover, elevated turbidity reduces water transparency, hindering the growth and reproduction of plankton and periphytic algae (Ma et al. 2021; Gao et al. 2024), thereby influencing the distribution of fish assemblages.

Water depth was also a key factor that affects fish distribution in the lower Yalong River. Species belonging to ‘lotic‐guild’ usually inhabit fast‐flowing and shallow water, whereas those belonging to ‘lentic guild’ can usually be detected in deeper and slower‐flowing water (Zhu et al. 2015). In our study, the ‘lotic‐guild’ mainly distributes in the tributaries of the Anning River, while the ‘lentic guild’ prefers the lower Yalong River (Tables 1 and 3). After the completion of the hydropower station, most of the lower Yalong River forms reservoir areas with high water levels and low velocity, causing the loss of the original fluviatile habitat and the change of sediment type (Ma et al. 2021). It results in the change of the river hydrological regime, the formation of a new water ecological environment, and the change of fish assemblages (Yang et al. 2021). The population stocks of *L* . *elongata* , *S* . *chongi* , and *P* . *pingi* decreased significantly after the construction of the hydropower station (Yang et al. 2011). In addition, the abundance of lotic fishes, including *C* . *guichenoti* and *R* . *ventralis* , decreased in the Jinping Bend of the Yalong River (Liu et al. 2022). In this study, the lower Yalong River was characterized by deep water, slow flows, fewer endemic lotic species, and more widespread lentic species like Cultrinae. Moreover, the mechanisms by which hydropower development influences fish distribution are complex and not fully understood. Therefore, additional research is necessary to examine the impact of dam operation modes on fish distribution in the lower Yalong River.

On the contrary, low‐head dams may have less impact on fish community structure than high‐head dams. Fish communities in the Anning River were more considerably affected by the natural conditions (including altitude and the substratum composition) than hydropower projects (Ma et al. 2023). Furthermore, based on the results of Fish index of biotic integrity (F‐IBI) evaluation, the health status of the Anning River was assessed as “average” and “good” level (Zhou 2024). As the greatest tributary in the lower Yalong River, the aquatic biodiversity of the Anning River exerts a vital role in maintaining ecosystem structure and function in the lower Yalong River (Ma et al. 2021). Therefore, some nature reserves can be established in the Anning River to protect indigenous lotic fishes.

## 5 Conclusions

To conclude, our results indicate that the lower Yalong River provides habitats for various endemic and endangered fish species. Fish species diversity increased first and then decreased, peaking in the mid‐order river, specifically the Anning River. Fish communities revealed significant variation with stream order. Nemacheilidae were predominant in the fish assemblages from the tributaries of the Anning River, while Gobioninae and Cultrinae were the most prevalent in the Anning River and the lower Yalong River, respectively. A turnover pattern was observed in the changes of key species in fish assemblages from low‐ to high‐order rivers. According to CCA, the water depth and turbidity were key factors for determining fish assemblages of the region. The findings provide a significant foundation for managing species biodiversity and conserving habitats for aquatic species in plateau waters. Furthermore, the results demonstrated that mid‐order rivers are crucial for sustaining the dendritic stream networks in mountain river systems.

## Author Contributions

**Huijun Ru:** formal analysis, methodology, writing – original draft. **Xiangxiang Wu:** formal analysis, investigation. **Bin Xu:** investigation. **Yan Zhang:** investigation. **Fan Wu:** formal analysis. **Baoshan Ma:** conceptualization, investigation, writing – review and editing.

## Conflicts of Interest

The authors declare no conflicts of interest.

## Supporting information


**Data S1:** ece371922‐sup‐0001‐DataS1.docx.

## Data Availability

The data that support the findings of this study are openly available in the Dryad Digital Repository at https://doi.org/10.5061/dryad.bnzs7h4n7.
