# Supplementary material for: Spatial Organization of Fish Assemblages and Its Relation to Environmental Factors in the Lower Yalong River, China
Source: Ecol Evol. 2025 Aug 7;15(8):e71922. doi: 10.1002/ece3.71922 (PMC12329763; doi:10.1002/ece3.71922)
Supplement: Supplementary file 1 — Data S1: ece371922‐sup‐0001‐DataS1.docx. [file ECE3-15-e71922-s001.docx]

**TABLE S1** Correlation coefficient of Spearman’s correlation analysis in the lower Yalong River and its tributaries

|  | **Water depth** | **Channel width** | **Current velocity** | **Water**  **temperature** | **Conductivity** | **Dissolved**  **oxygen** | **pH** | **Turbidity** | **Boulder** | **Cobble** | **Pebble plus gravel** | **Sand** | **Silt plus clay** |
| --- | --- | --- | --- | --- | --- | --- | --- | --- | --- | --- | --- | --- | --- |
| Altitude | -0.532 | -0.649* | 0.177 | -.836* | -0.770* | -0.035 | -0.177 | -0.15 | 0.251 | -0.043 | -0.332 | -0.359 | 0.066 |
| Water depth |  | 0.776* | -0.588 | 0.428 | 0.804* | 0.355 | 0.352 | -0.246 | -0.614* | -0.112 | 0.226 | 0.503 | 0.189 |
| Channel width |  |  | -0.578 | 0.610* | 0.820* | 0.297 | 0.299 | -0.075 | -0.642* | 0.151 | 0.344 | 0.369 | 0.207 |
| Current velocity |  |  |  | -0.142 | -0.43 | -0.487 | 0.014 | 0.292 | 0.746* | 0.108 | -0.412 | -0.343 | -0.415 |
| Water temperature |  |  |  |  | 0.678* | -0.186 | 0.144 | 0.204 | -0.257 | 0.046 | 0.378 | 0.124 | 0.174 |
| Conductivity |  |  |  |  |  | 0.15 | 0.374 | -0.084 | -0.421 | 0.126 | 0.369 | 0.359 | 0.043 |
| Dissolved oxygen |  |  |  |  |  |  | 0.002 | -0.424 | -0.277 | 0.197 | 0.104 | 0.121 | -0.024 |
| pH |  |  |  |  |  |  |  | -0.028 | -0.218 | 0.106 | -0.03 | 0.281 | -0.038 |
| Turbidity |  |  |  |  |  |  |  |  | -0.122 | -0.027 | 0.11 | 0.244 | 0.333 |
| Boulder |  |  |  |  |  |  |  |  |  | 0.223 | -0.501 | -0.659* | -0.585 |
| Cobble |  |  |  |  |  |  |  |  |  |  | 0.049 | -0.461 | -0.374 |
| Pebble plus gravel |  |  |  |  |  |  |  |  |  |  |  | 0.197 | 0.154 |
| Sand |  |  |  |  |  |  |  |  |  |  |  |  | 0.323 |

Note: ^*^. Correlation coefficients > 0.6
